# Efficacy of catheter interventions in the early and very early postoperative period after CHD operation

**DOI:** 10.1017/S1047951118001452

**Published:** 2018-09-03

**Authors:** Takuro Kojima, Tomohiko Imamura, Yousuke Osada, Shouta Muraji, Marie Nakano, Takayuki Oyanagi, Shigeki Yoshiba, Toshiki Kobayashi, Naokata Sumitomo

**Affiliations:** Division of Pediatric Cardiology, Saitama Medical University International Medical Center, Saitama, Japan

**Keywords:** Catheter intervention, very early postoperative period, balloon angioplasty, stent implantation, intervention for the left-sided heart

## Abstract

**Background:**

Catheter interventions for residual lesions in the early postoperative period after CHD operations are still not established as a reliable treatment option.

**Methods:**

We retrospectively reviewed our institutional experience of cardiac catheterisations and catheter interventions performed in the early postoperative period. We classified our patients into two groups. The “hyper” acute phase group – operation to cardiac catheterisation of ⩽7 days – and acute phase group – operation to cardiac catheterisation from 7 to 30 days.

**Results:**

Of the 47 patients, catheter interventions were performed in 38 patients (81%). The success rate of the intervention was 96% in the acute phase group and 90% in the “hyper” acute phase group. The overall success rate was 95%. There were two self-limited complications in the acute phase group, but not in the “hyper” acute phase group. There were four cases of catheter interventions performed for a newly reconstructed aortic arch, and those procedures were also safe and effective.

**Conclusions:**

Cardiac catheterisations and catheter interventions were safe and effective not only in the early postoperative period but also in the very early postoperative period. Catheter interventions for the left-sided heart in the early postoperative period were also safe and effective.

Over the past few decades, transcatheter interventions have become an important treatment option for children with CHD.^1^ ^–^ ^3^ Currently, the procedures may be performed safely and effectively, even in small infants or neonates.^4^ On the other hand, it has been considered that cardiac catheterisations and catheter interventions in the early postoperative period after CHD operations are associated with excessive risks. These risks include transporting critically ill children to the catheterisation laboratory and anxiety for disruption of fresh surgical suture lines.^5^ Therefore, cardiac catheterisations and catheter interventions for residual lesions in the early postoperative period after CHD operations are still not established as a reliable treatment option. In the present study, we reviewed our institutional experience with patients who received cardiac catheterisations and catheter interventions in the early postoperative period and attempted to reveal the safety and efficacy of the procedures in these patients.

## Materials and methods

### Study patients

This retrospective study was approved by the Institutional Review Board of Saitama Medical University International Medical Center. This study included 47 children – 29 boys and 18 girls, with a mean age of 6 months [day 14–7 years] – who underwent cardiac catheterisation in the early postoperative period (⩽30 days) after a CHD operation at the International Medical Center, Saitama Medical University, between July, 2011 and April, 2017.

### Catheter procedures and the indication for catheter intervention

All the catheter procedures were performed under general anaesthesia in the catheterisation laboratory. The anaesthesia was performed by a paediatric cardiac anaesthesiologist, and all the procedures were performed by paediatric interventional cardiologists. The procedures included balloon angioplasty, stent implantations, coil embolisations, and balloon atrial septostomy. The indications for postoperative cardiac catheterisation included extracorporeal membrane oxygenators with withdrawal difficulty, closed-chest difficulty, tracheal extubation difficulty, and morphological problems revealed by imaging – echocardiography and/or chest CT scan.

### Assessment of procedural success

Interventional procedures were considered as successful or unsuccessful by previously cited criteria. The criteria included (1) angioplasty and stent implantations: a vessel diameter increase of >75% and/or a >50% reduction in the peak systolic pressure gradient across the stenosis,^6^ (2) vascular occlusions: no residual flow on angiography, and (3) balloon atrial septostomy: non-restrictive interatrial flow by echocardiography. In addition, in this study, we used the clinical criteria for procedural success. The clinical criteria included avoidance of re-operations for residual lesions, withdrawal from the extracorporeal membrane oxygenators, reached the closed-chest stage, and reached the next surgical stage. Finally, we assessed the survival rate. In this study, we defined the “survival” as discharge from the hospital. We compared the survival rate of patients who underwent catheter interventions and patients who did not undergo catheter interventions and assessed whether the catheter interventions for those patients led to better survival.

### Time from the predecessor operation

In this study, we classified the patients into two groups according to the time from the predecessor operation to the cardiac catheterisation. These two groups were the “hyper” acute phase group – time from the operation to the cardiac catheterisation of ⩽7 days – and acute phase group – time from the operation to the cardiac catheterisation from 7 to 30 days.

### Statistical analysis

All continuous data are expressed as mean±SD. Comparisons between the two groups were performed using a paired or an unpaired t test or Fisher’s exact test. Vessel diameters before and after the procedure were compared using a paired t test. A p<0.05 was considered statistically significant. All statistical analyses were performed using GraphPad PRISM version 7.02 (GraphPad Software Inc., La Jolla, California, United States of America) software.

## Results

### Patient characteristics

In all, 47 cardiac catheterisations were performed in the study period. The median age at the time of the catheterisation was 6 months (day 14–7 years), and the time from the predecessor operation to the cardiac catheterisation was 14.2 days (1–30 days).

Table 1 shows the comparison of the patient characteristics between the “hyper” acute phase group and acute phase group. Patients in the “hyper” acute phase group tended to be younger compared with the acute phase group, although it was not statistically significant (3.1±0.9 and 7.5±2.7 months, respectively, p=0.31). The rate of cardiac catheterisation and catheter interventions performed under extracorporeal membrane oxygenator administration was significantly higher in the “hyper” acute phase group than that in the acute phase group (43 and 3%, respectively, p=0.002). Although the total survival rate was slightly higher in acute phase group (79 and 64%, respectively), the survival rate in those who underwent catheter intervention was higher in the “hyper” acute phase group than that in the acute phase group (90 and 82%, respectively). There were two catheter-related complications in the acute phase group: pulmonary haemorrhage and haematochezia. Both complications were self-limited and did not need any additional medications. In the “hyper” acute phase group, on the other hand, there were no catheter-related complications.

**Table 1:** Patient characteristics.

n=47	“Hyper” acute group (n=14)	Acute group (n=33)	p Value
Age, month	3.1± 0.9	7.5±2.7	0.31
Interventions, %	71	85	0.42
ECMO, %	43	3	0.002
Complications, n	0	2	0.9
Total survival, %	64	79	0.46
Survival after intervention, %	90	82	0.9

ECMO=extracorporeal membrane oxygenators

### Diagnosis and the predecessor operations

Table 2 shows the primary diagnoses of the patients. The range of primary diagnoses was heterogeneous, but the majority had complex congenital anomalies, and more than half had single ventricles and hypoplastic left heart syndrome. Table 3 summarises the type of operations before the cardiac catheterisations or catheter interventions. The aortopulmonary shunts and Norwood operation+aortopulmonary shunts/bidirectional Glenn anastomosis accounted for more than half of the entire operations.

**Table 2:** Primary diagnosis.

	n	%
Single ventricle	13	27.8
Hypoplastic left heart syndrome	12	25.7
Transposition of the great arteries	4	8.5
Tetralogy of Fallot	3	6.4
Interrupted aortic arch	3	6.4
Double-outlet right ventricle	2	4.2
Complete atrioventricular septal defect	2	4.2
Tricuspid valve atresia	2	4.2
Coarctation of the aorta	2	4.2
Others	4	8.4

**Table 3:** Type of predecessor operation.

Type of operation	n
Aortopulmonary shunt	10
Norwood operation and aortopulmonary shunt	9
Norwood operation and BDG	8
BDG	4
Arterial switch operation	4
Repair of tetralogy of Fallot	3
Hybrid stage 1 operation	3
Others	6

BDG=bidirectional Glenn anastomosis

### Catheter interventions

**Figure 1:**
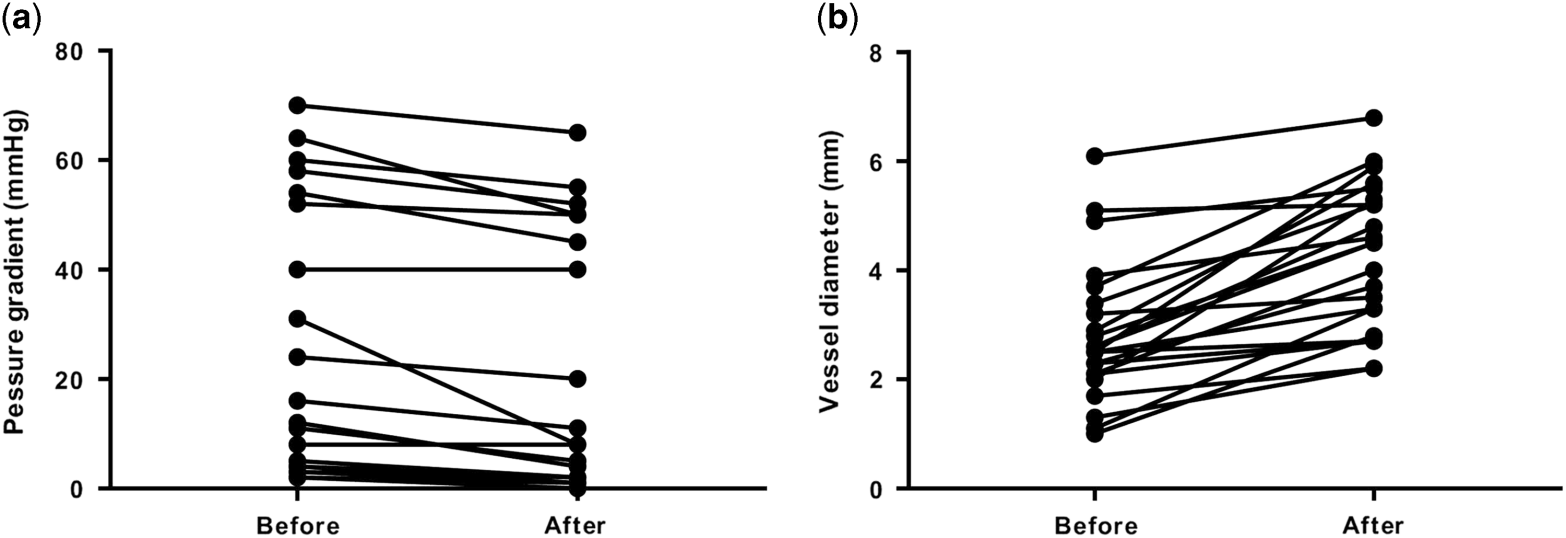
Pressure gradients and vessel diameter before and after balloon dilatation. (***a***) Pressure gradients before and after balloon dilatation. (***b***) Vessel diameter before and after balloon dilatation.

**Figure 2:**
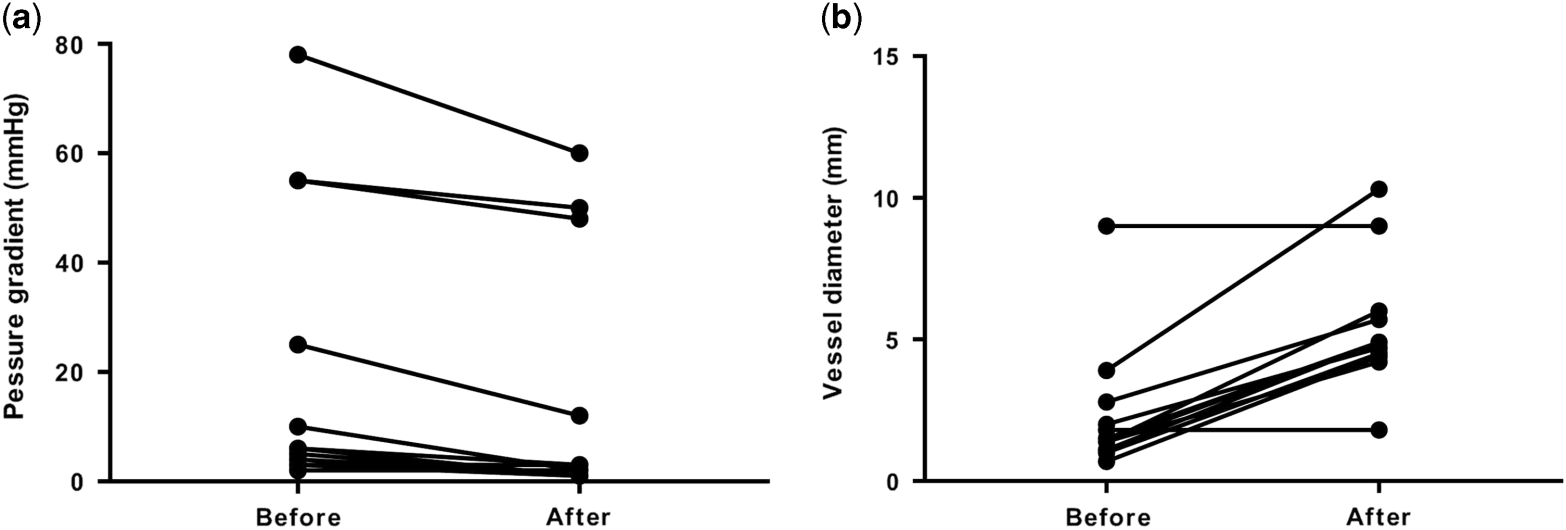
Pressure gradients and vessel diameter before and after stent implantation. (***a***) Pressure gradients before and after stent implantation. (***b***) Vessel diameter before and after stent implantation.

Of the 47 patients who underwent cardiac catheterisations, catheter interventions were performed in 38 patients (81%). Catheter interventions were not performed in the remaining nine patients because there were no morphological problems including stenotic lesions requiring catheter interventions in those patients. Among the residual lesions, the left pulmonary artery was the most common (n=18), followed by the right pulmonary artery (n=5) and aortopulmonary shunt (n=5). Most of these residual lesions were stenotic lesions. It is notable that, there were four cases in whom catheter interventions were performed for an aortic arch. All four cases belonged to the acute phase group (9–27 days). The procedures included two cases of balloon angioplasty for a re-coarctation after a repair of a coarctation of the aorta, one case of balloon angioplasty after a Norwood operation and aortopulmonary shunt, and one case of a stent implantation for an aortic dissection after a hybrid stage 1 operation for hypoplastic left heart syndrome. Table 4 shows the type of procedures. Balloon angioplasty was the most common, followed by stent implantations. Although most of the stenotic lesions were released effectively by angioplasty, some stenotic lesions were formed by compression from the surrounding structures; most of these lesions were the left pulmonary arteries compressed by a newly reconstructed aortic arch. For those lesions, stent implantations were safe and effective compared with balloon angioplasty. After the balloon dilation, the pressure gradient across the stenotic lesion was significantly decreased (p=0.0004, Fig 1a). The vessel diameter after the balloon dilation was significantly larger than that before the procedure (p<0.0001, Fig 1b). In addition, after the stent implantation, the pressure gradient across the stenotic lesion was significantly decreased (p=0.004, Fig 2a). The vessel diameter after the stent implantation was significantly larger than that before the procedure (p<0.0001, Fig 2b). Similarly, the interatrial pressure gradient and size of the interatrial defect before and after the procedure were assessed. Although the number of patients was small (n=2), balloon atrial septostomy was also effective in these patients. The pressure gradient decreased from 8 to 3 mmHg and 6 to 2 mmHg, respectively. The interatrial defect had enlarged from 2.2 to 5.0 mm and 2.0 to 5.2 mm, respectively. The intervention success rate was 96% in the acute phase group, 90% in the “hyper” acute phase group, and the overall success rate was 95%.

**Table 4:** Type of procedures.

Procedures	n
Balloon angioplasty	23
Stent implantation	12
Balloon atrial septostomy	2
Coil embolisation	1

The total survival rate – discharged from the hospital – in the patients who underwent catheter interventions was higher than that in those who did not undergo catheter interventions (84 and 33%, respectively).

### Procedures performed under extracorporeal membrane oxygenators administration

There were seven cases of cardiac catheterisations and catheter interventions performed under extracorporeal membrane oxygenators administration. Of the seven patients, catheter intervention was performed in three patients and two of them were able to withdraw from extracorporeal membrane oxygenator administration. All patients who did not undergo catheter intervention died.

## Discussion

Catheter interventions have become an important option in the treatment of postoperative residual lesions. Improvements in the catheter devices and techniques have allowed the application of many of these techniques even in small infants or neonates.^4^ Nowadays, there are some reports regarding the safety and efficacy of catheter interventions in the early postoperative period.^7^ ^,^ ^8^ However, cardiac catheterisations and catheter interventions for residual lesions in the early postoperative period after CHD operations are still not established as a reliable treatment option because of anxiety of its excessive risk, including transporting these unstable children to the catheterisation laboratory and fear of disruption of fresh surgical suture lines. Reports of the safety and efficacy of cardiac catheterisations and catheter interventions in the very early postoperative period are much more limited. In the present study, we reviewed our experience of cardiac catheterisations and catheter interventions in the early postoperative period after CHD operations. One of the striking findings in the study was that catheter interventions were performed safely and effectively not only in the early postoperative period but also in the very early postoperative period. In fact, patients who underwent catheter interventions in the very early postoperative period yielded a high intervention success rate without any catheter-related complications. In the past, it was thought that at least 6 weeks were necessary to perform catheter interventions safely after CHD operations. This was owing to the opinion that such a period was necessary for adequate formation of scar tissue around surgically anastomosed sites. However, in the present study, although patients who underwent catheter intervention in the very early postoperative period were younger, they showed a high intervention success rate without intervention-related complications. This suggested that a shorter postoperative period or smaller age did not necessarily exclude the chance of performing cardiac catheterisations or catheter interventions after CHD operations.

In the present study, most of the patients suffered from an unbalanced pulmonary circulation and decreased pulmonary flow, which might have led to extracorporeal membrane oxygenators with withdrawal difficulty, closed-chest difficulty, and tracheal extubation difficulty. After early and very early postoperative catheter interventions, the pulmonary circulation improved with an increased pulmonary flow. This might have led to a better outcome than in patients who did not undergo catheter interventions. In addition, we found that there were four cases in whom the catheter interventions were performed for an aortic arch. Although it was considered that the catheter intervention for the left-sided heart after a CHD operation was especially risky, the procedures were performed safely without any complications. In the present study, we did not have data on the safety of performing catheter intervention for the left-sided heart in the very early postoperative period. However, we considered that postoperative periods of 10–14 days are sufficient to perform catheter interventions for the left-sided heart.

In the present study, there were seven cases of cardiac catheterisations and catheter interventions performed under extracorporeal membrane oxygenators administration. There are several reports that have described the safety of performing cardiac catheterisations or catheter interventions in children under extracorporeal membrane oxygenator administration.^9^ ^–^ ^11^ However, the results are yet unsatisfactory. In this study, the overall survival rate of patients who underwent cardiac catheterisation or catheter intervention under extracorporeal membrane oxygenator administration was 29%, and the survival rate in those who underwent catheter intervention was 50%. All the patients who did not undergo a catheter intervention died. Therefore, the prognosis improvement of cardiac catheterisation or catheter intervention under extracorporeal membrane oxygenator administration is still a problem to be resolved.

In conclusion, we found that cardiac catheterisation and catheter intervention in the early and very early postoperative periods after CHD operations was safe and effective. In addition, catheter interventions for the left-sided heart in the early postoperative period were also safe and effective. We expect that this aggressive strategy will yield a better prognosis for extremely sick children who underwent CHD operations.
